# How Do Online Social Networks Grow?

**DOI:** 10.1371/journal.pone.0100023

**Published:** 2014-06-18

**Authors:** Konglin Zhu, Wenzhong Li, Xiaoming Fu, Jan Nagler

**Affiliations:** 1 Institute of Computer Science, Georg-August-Universität Göttingen, Göttingen, Germany; 2 State Key Laboratory for Novel Software and Technology, Nanjing University, Nanjing, China; 3 Computational Physics, IfB, ETH Zurich, Zurich, Switzerland; 4 Max Planck Institute for Dynamics and Self-Organization (MPI DS), Göttingen, Germany; Max Planck Institute for the Physics of Complex Systems, Germany

## Abstract

Online social networks such as Facebook, Twitter and Gowalla allow people to communicate and interact across borders. In past years online social networks have become increasingly important for studying the behavior of individuals, group formation, and the emergence of online societies. Here we focus on the characterization of the average growth of online social networks and try to understand which are possible processes behind seemingly long-range temporal correlated collective behavior. In agreement with recent findings, but in contrast to Gibrat's law of proportionate growth, we find scaling in the average growth rate and its standard deviation. In contrast, Renren and Twitter deviate, however, in certain important aspects significantly from those found in many social and economic systems. Whereas independent methods suggest no significance for temporally long-range correlated behavior for Renren and Twitter, a scaling analysis of the standard deviation does suggest long-range temporal correlated growth in Gowalla. However, we demonstrate that seemingly long-range temporal correlations in the growth of online social networks, such as in Gowalla, can be explained by a decomposition into temporally and spatially independent growth processes with a large variety of entry rates. Our analysis thus suggests that temporally or spatially correlated behavior does not play a major role in the growth of online social networks.

## Introduction

Online social networks (OSNs) have become increasingly important as they allow us to interact across any geographical scale. Communication networks, transport networks and OSNs are often interconnected and interdependent. This opens up great economic and social opportunities but can also involve considerable risks such as cascading breakdowns [1]. The study of OSNs is of importance for understanding the behavior of individuals, groups and societies. Hence, particular types of growth in social, economic and other networked systems have attracted a lot of attraction in the past years [2]–[8].

Gibrat's law states that both the average growth rate and the standard deviation of the growth rate of a given observable are constant and independent of the specific value of the observable [9]. However, this empirical law, originally observed in economic systems, has been challenged by many socio-economic studies [10], [11], notably very recently [4], [5], [8].

Any social growth dynamics is expected to depend on social factors such as gender, age, social status and so forth. Unfortunately, available datasets that comprise such information are typically too small to investigate emergent scaling or large-scale collective behavior. In this paper, we focus on the population growth dynamics of three large OSNs. Our datasets do not resolve individual social factors but their size allows for studying scaling and long-range correlations, both temporally and spatially.

We find evidence for certain scaling laws in the growth rate and the variance, although for Renren and Twitter the exponents characterizing fluctuations are found to deviate from those that have been reported previously for social and economic systems. These deviations carry important information about the growth of online social systems. In particular, we find that the relative number of registered users increases almost temporally and spatially independently of each other. This contrasts the behavior of offline growth in many social and economic systems where growth is a long-range correlated process and thus a collective phenomenon. Even for Gowalla where scaling indicates long-range correlated growth a decomposition into independent growth processes unravels the seemingly long-range collective behavior to be a mere artifact of the large variability of entry rates [12].

## Data

We analyze three OSN datasets. The first OSN Renren (rr), often referred to as the “Chinese Facebook”, is one of the largest online social networks in China. The dataset covers about 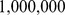 users in the time period of January 2006 to December 2010 (60 months) with online interactions from over 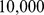 registered locations.

The second OSN data set, comes from a subset of Twitter (tw), a microblogging online social service sited in the United States. It covers more than 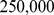 members between August 2006 and September 2010 (50 months) from about 9,000 locations.

The third OSN, Gowalla (gw), was an online check in social service launched in 2007 and closed in 2012 in the United States. Users were able to *check in* at certain locations, referred to as *Spots*, either through a mobile phone application or Gowalla's mobile website. Among other things, checking-in allowed for the dropping or swapping of virtual items. The dataset covers 21 months (from February 2009 to October 2010) with around 200,000 members from about 5,000 locations.

We acquired the first two datasets by crawling user profiles in the web sites from Renren.com [13] and Twitter.com [14] through their APIs. We only crawled the user profiles which are publicly available. The Gowalla dataset is obtained from a shared data source [15] by other researchers. Due to the tremendous size of OSNs, we only acquired a sampled subset of each OSN. To eliminate sample bias we deployed the Breadth First Search (BFS) bias correction procedure by Kurant et al. [16].

For these three datasets we define a *population* at a location 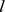 (an integer number ID) at time 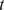 as the set of all users with home location 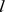. The spatial resolution of the location refers to as a city code, associated with the administrative area (i.e. the city name) of the user's home location. For Renren and Twitter we assume the registered location of the user as the user's home location. For Gowalla we assume the most visited location as the home location. For the spatial analysis we used an assignment of GPS coordinates (via Google Maps Application Programming Interfaces (API)) to the location 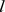, and calculated the distance between two locations via their GPS coordinates. The estimated GPS coordinates of a user's home location may thus be incorrect for a certain fraction of a given population. This, however, may not alter any of the conclusions made in this article.

## Results

Here, we investigate the mean growth rate and its fluctuation in OSN populations and ask the question how these observables depend on the initial population size.

We denote the population size, i.e. the number of users with home location index 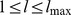 at time 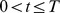, by 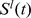. Following Refs. [17]–[19] we define the logarithmic growth rate 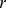 between time 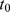 and 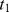 (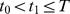) as
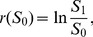
(1)where 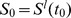 and 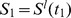 are the population size at a location 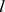 but at different time points 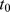 and 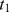 [5].

**Figure 1:**
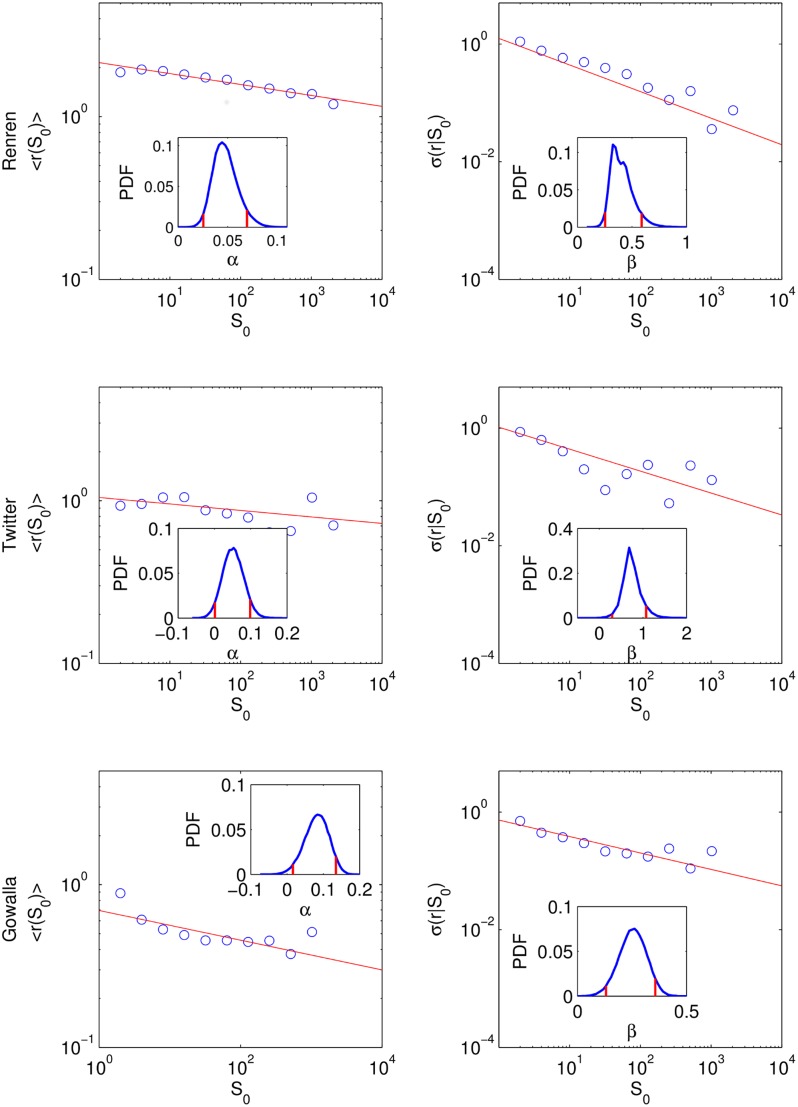
Scaling in average growth rate and standard deviation. Both 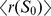 and 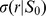 as a function of the initial population size 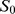 exhibit a power law, 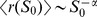, 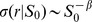. Renren: 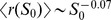 (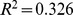), 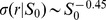 (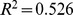), that is, 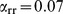, 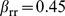, Twitter: 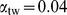 (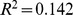), 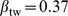 (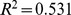), Gowalla: 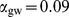 (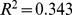), 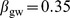 (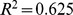). All values are obtained from MLE. Bootstrapping suggests 95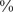 confidence for 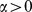 (violation of Gibrat's law), and for 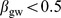 (suggesting long-rang correlations). No statistical significance is found for 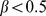 for Renren and Twitter. Vertical lines indicate 5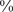 marks (insets).

To characterize fluctuations, we study the average growth rate 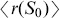 and the standard deviation
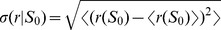
(2)as a function of the initial population size 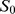, see Figure 1. In other words, the average growth rate 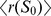 corresponds to only those online populations with size at least 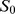 until time 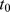. The conditional standard deviation of the growth rate 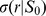 for those populations expresses the statistical spread or fluctuation of growth among populations with 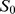. Both quantities show a power law dependence on the initial population size,
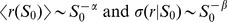
(3)with positive exponents 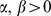 (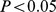), which suggests a deviation from the independence of Gibrat's law that would imply 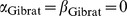. Scale-invariant growth instead of Gibrat's proportionate growth has been reported for economic systems such as firms [10] and countries (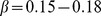) [20], research and development expenditures at universities (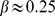) [21], scientific output (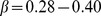) [22], and more recently for city population growth (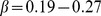) [4] and online communities (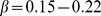) [5].

The range of 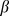 for Renren and Twitter is in agreement with those previously reported exponents for 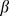. However, in contrast to the previous work mentioned above our analysis (employing maximal likelihood estimation (MLE) and bootstrapping) do not suggest significant deviations from 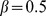 which would indicate uncorrelated growth. In contrast, for Gowalla we find 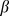 significantly smaller than 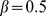 (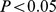).

Second, we find the range of exponents for the average growth rate for all studied online social networks significantly above 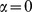 (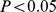) indicating a violation of Gibrat's proportionate growth, which is in agreement with social and economic systems [4], [5], [10], [20]–[22].

The average growth rate 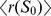 and the conditional standard deviation of the growth rate 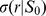 allow for direct comparison with the literature for other social systems and Gibrat's law but are only averages. As suggested by studies of certain assets in economical systems the distribution of the variance can often exposes important information that cannot be seen in averages [23].

**Figure 2:**
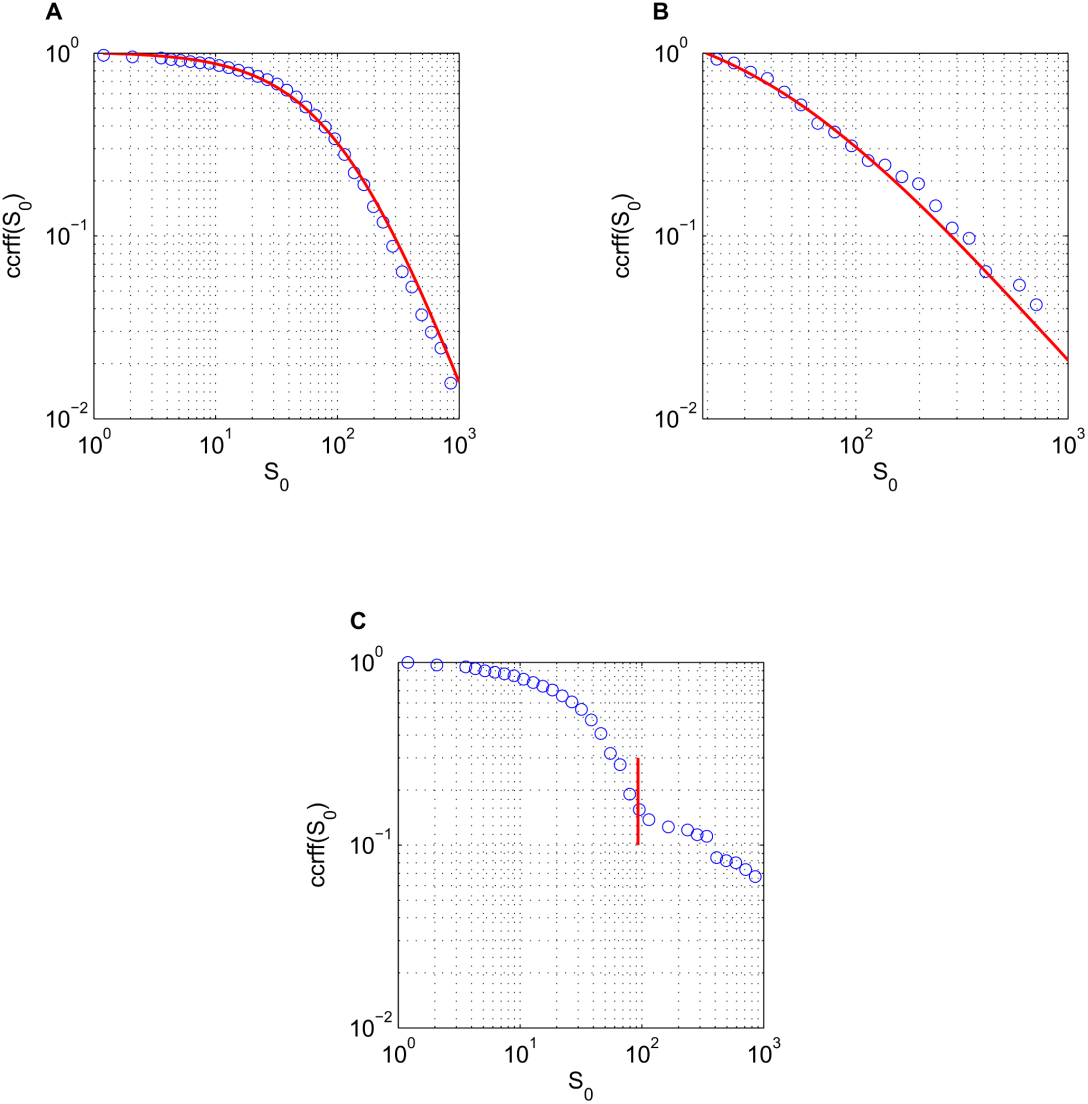
Complementary cumulative relative fluctuation function. The ccrff, equation (5), as a function of the initial population size 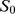. For Renren and Twitter the ccrff is well fitted by shifted power law 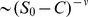, with 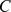 a constant: (a) Result for Renren: 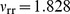 (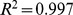). (b) Result for Twitter: 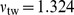 (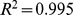). (c) For Gowalla the ccrff is bimodal with a cutoff point at 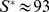 (obtained from MLE, see methods): the left part is well fitted by an exponential and the right part is in good agreement with a power law decay, ccrff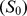 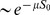 for 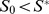, and ccrff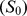 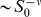 for 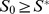. Fit exponents 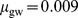 (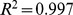), and 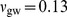 (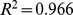).

Since for a given 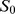 there is only a single value of 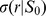 (see Fig. 1), we ask what is the relative variation of 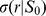 across all values of 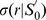 that occur in a given dataset. We thus focus on the relative fluctuation function (rff),
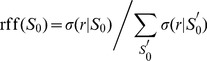
(4)as a function of 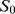. Specifically, we study the complementary cumulative relative fluctuation function (ccrff), which is given by the complement of the integrated rff,
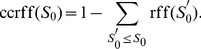
(5)We chose the ccrff representation because it shows (if exists) a clearer scaling than the rff and thus better exposes different (scaling) regimes. The ccrff is obtained by collecting all locations with a given value of 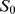 using exponential binning (see Fig. 2 and Methods).

In contrast to Renren and Twitter where we find no significant bimodality, for Gowalla the ccrff as a function of 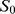 exhibits a remarkable bimodal behavior.

For Gowalla, Figure 2C suggests a bimodal distribution of standard deviations, characterized by an exponential decay that is followed by a power law
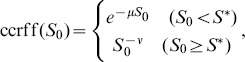
(6)

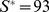
 for Gowalla marks the crossover point (determined from MLE, see Methods). MLE suggests that the power law decay is characterized by the exponent 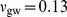 (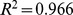).

### Gowalla: Correlations in the growth rate

**Figure 3:**
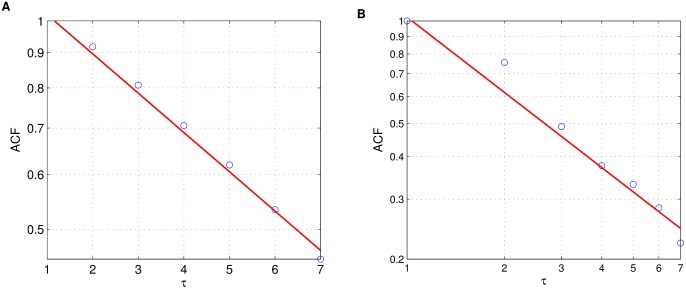
Gowalla: Temporal short- and long-term correlations in the population growth rate. Short-term correlations for 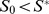 (log-lin plots), 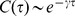, and long-term correlations for 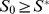 (log-log plots), 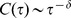. Fits using MLE suggest 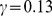 (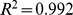), 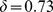 (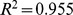); log-log-scaling for determining the coefficient of determination for the power law, and log-linear-scaling for the exponential.

The above findings suggest to consider two groups of locations: one group with initial population size 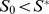, and the other one with initial population size 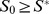. We study the monthly population growth rates for each location and calculate their autocorrelation function (ACF) [24]–[26]. For 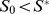 the ensemble averaged ACF exhibits an exponential decay, 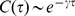, indicating that the population growth is short-term correlated, see Figure 3A. We obtain the exponent 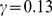 (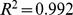 from MLE), which is equivalent to a correlation time constant of about two weeks.

In contrast, for 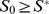 the ACF is well described by a power law, 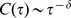 with power law exponent 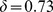 (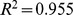 from MLE), see Figure 3B.

This is consistent with long-term correlations characterized by 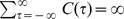, see [27] and references therein.

### Superposition model

Seemingly long-range correlation can often be explained by a finite set of independent processes whose superposition accounts for the algebraic decay in the ACF, and the divergence of its infinite sum. In 1979 van der Ziel established that any ensemble of uncoupled short-range correlated stochastic oscillators is sufficient for explaining long-range correlations in their superposition, if and only if the time constants of the mixed processes are sufficiently broadly distributed [12]. More recently, it has been shown that a superposition of Poisson processes, together with circadian activity, very likely account for many scaling laws of human activity patterns [28]. Here, as the growth rates are broadly distributed, we follow this spirit by considering a superposition of populations and surrogate time series from these, see Methods.

**Figure 4:**
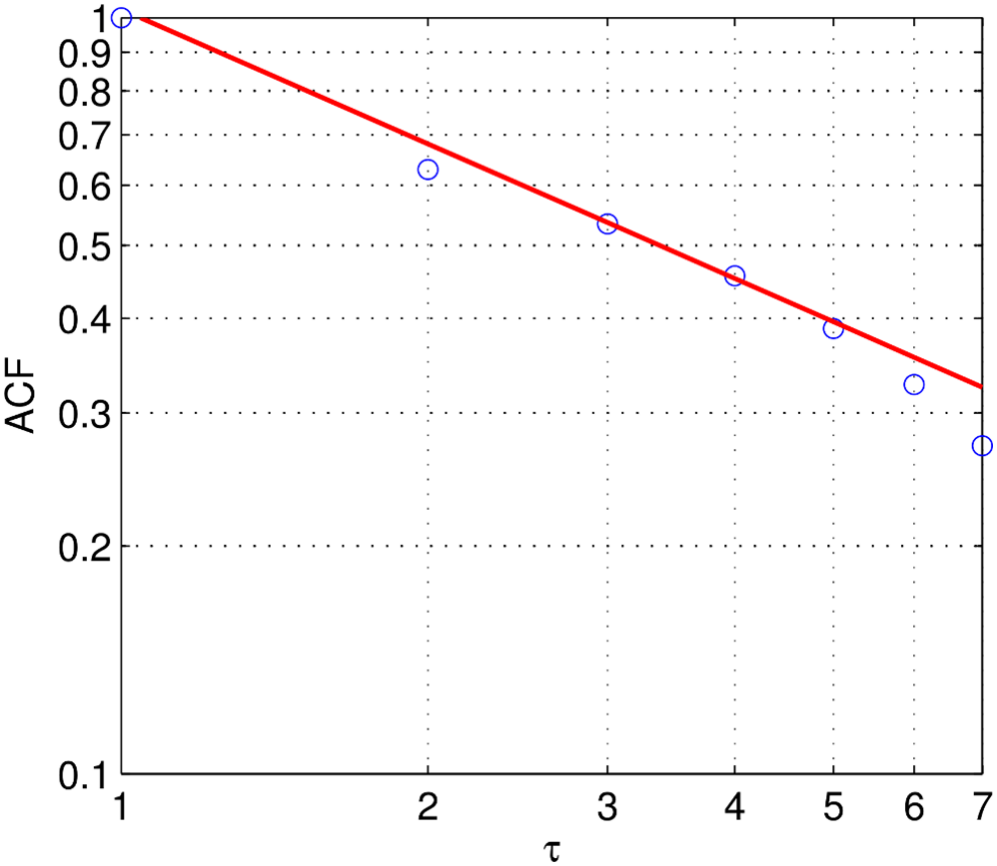
Gowalla: Decomposition of the growth into independent short-term correlated population growth processes. ACF for the three data sets according to the superposition scheme explained in the text. The power law exponents from fitting 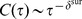 obtained from the decomposition via surrogate data (sur). Best fits from MLE: 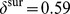 (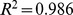).

Gowalla's population growth of the superposition ensemble obtained from a random selection of population with 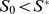 results in the occurrence of seemingly long-term correlations for locations with 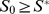. The exponents 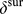 for the surrogate superpositions (sur) are obtained from fitting the superposition ensemble averaged ACF by MLE (see Methods) with 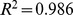, 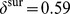 (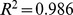, 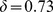), see Figure 4.

This suggests that the seemingly long-term correlated population growth found for locations with 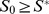 results from superpositions of short-term correlated growing populations.

### Spatial dependence

To study geographical factors we investigate correlations of the populations growth rates 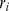 and 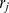 between different places [29], [30].

We therefore study the Pearson's correlation coefficient
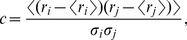
(7)where 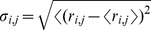 is the standard deviation of 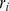 and 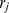, respectively.

**Figure 5:**
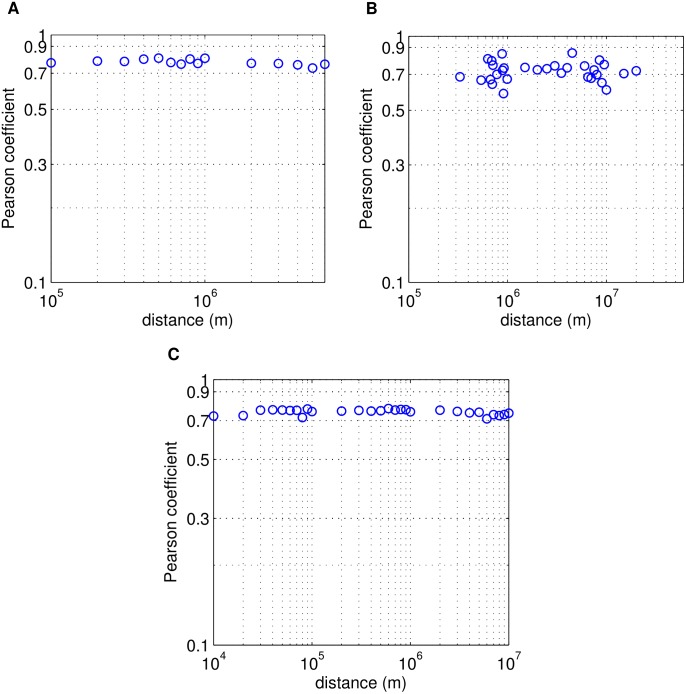
Spatial independence of the population growth rates. The mean correlation coefficient 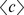 of the population growth rate as a function of geographical distance (log-log plot). (a) for Renren: plateau at about 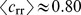, (b) for Twitter: 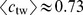, (c) for Gowalla: 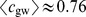.

We investigate the monthly population growth rates and Pearson's correlation coefficient between a pair of locations as a function of the geographic distance of the users. Figure 5 shows the Pearson's correlation coefficients for the three data sets. The average correlation 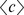 is found at a level of about 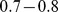, effectively independent of the geographic distance. The high value of the cross correlation agrees well with the plausible assumption that individuals join online social networks collectively but independently of the geographic distance to each other.

## Discussion

We find scaling in the population growth rate and variance in online social networks. Our results suggest that the population growth in online social networks is neither significantly determined by population size [31] nor by spatial factors. The results deviate from Gibrat's law as previously found in many social and economic systems. The seemingly long-term correlated growth behavior for Gowalla suggested by scaling in the standard deviation is explained by a simple decomposition into short-term correlated population growth with broadly distributed growth rates. Our method may help interpreting (seemingly) long-range correlations in the growth of large heterogenous (online) social and economic systems. Seemingly collective behavior in online social systems may result from the high variability of loners' actions and not from correlated collective behavior.

## Methods

### Ethics statement

We use the APIs that provided by Renren.com and Twitter.com for data collection from these two websites. The acquirement of Renren and Twitter datasets is in accordance with the websites' terms of service.

### Data availability statement

We use three datasets in this article. The Renren and Twitter datasets can be obtained upon the request, which is “data available on request”. The request can be send to the Computer Networks Group at University of Göttingen via email (net@cs.uni-goettingen.de). The Gowalla dataset is obtained from a shared data source [15] by other researchers. The requester can download it from snap.stanford.edu, which is “data available from online”.

### Exponential binning

Fitting of average, standard deviation and the ccrff is performed by exponential binning, by which the bins are evenly distributed on a logarithmic scale. Specifically, the beginning of each bin is 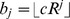, exponentially increasing in 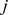, with constants 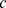 and 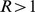, so that bins have size 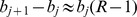. We use exponential binning for both Figure 1 (with 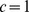, 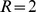) and Figure 2 (with 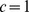, 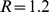).

### Choice of 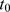 and 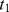


**Figure 6:**
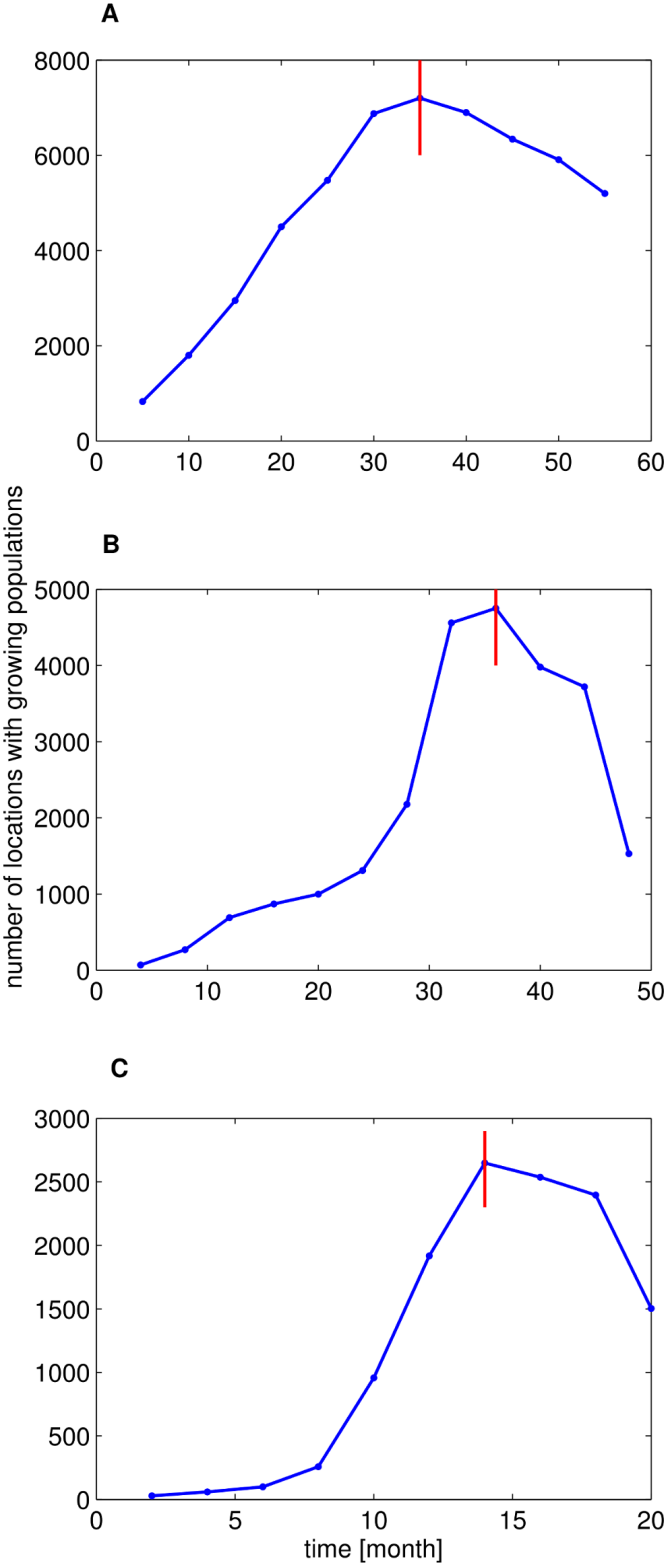
Selection method for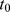. The number of locations with growing populations (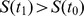) as a function of time. We account for the tradeoff between a large number of populated locations and a large time series length 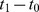 by choosing 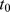 close to the maximum, cf. Methods. This results (a) for Renren to 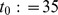, (b) for Twitter to 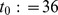, and (c) for Gowalla to 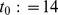.

The datasets are analyzed within a time window given by the time points 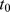 and 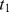. 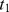 is chosen as the end point of the data set, 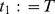. For the choice of 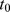 we consider two factors: the number of populated locations in the time window and the size of the time window. A too small 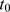 would lead to only a few populated locations whereas any large 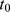 would reduce the width of the window. Following the methodology of studies in human population growth in the real world [4] and the human interaction activities in OSNs [5], we determined 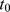 as a result from time when the number of locations with growing populations reaches the peak. That is, 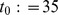 for Renren, 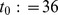 for Twitter and 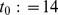 for Gowalla, respectively, see Figure 6.

### Determination of 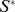


To determine the best value for 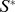, we fit the distribution of standard deviation with respect to 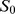 ranging from 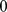 to 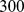 by using MLE. For each 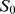, we calculate 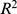 for exponential and power-law fitting, denoted as 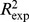 and 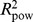, respectively. To characterize the overall fitting quality (FQ) we use
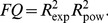
(8)where we use log-log-scaling for determining the coefficient of determination for the power law, and log-linear-scaling for the exponential.

**Figure 7:**
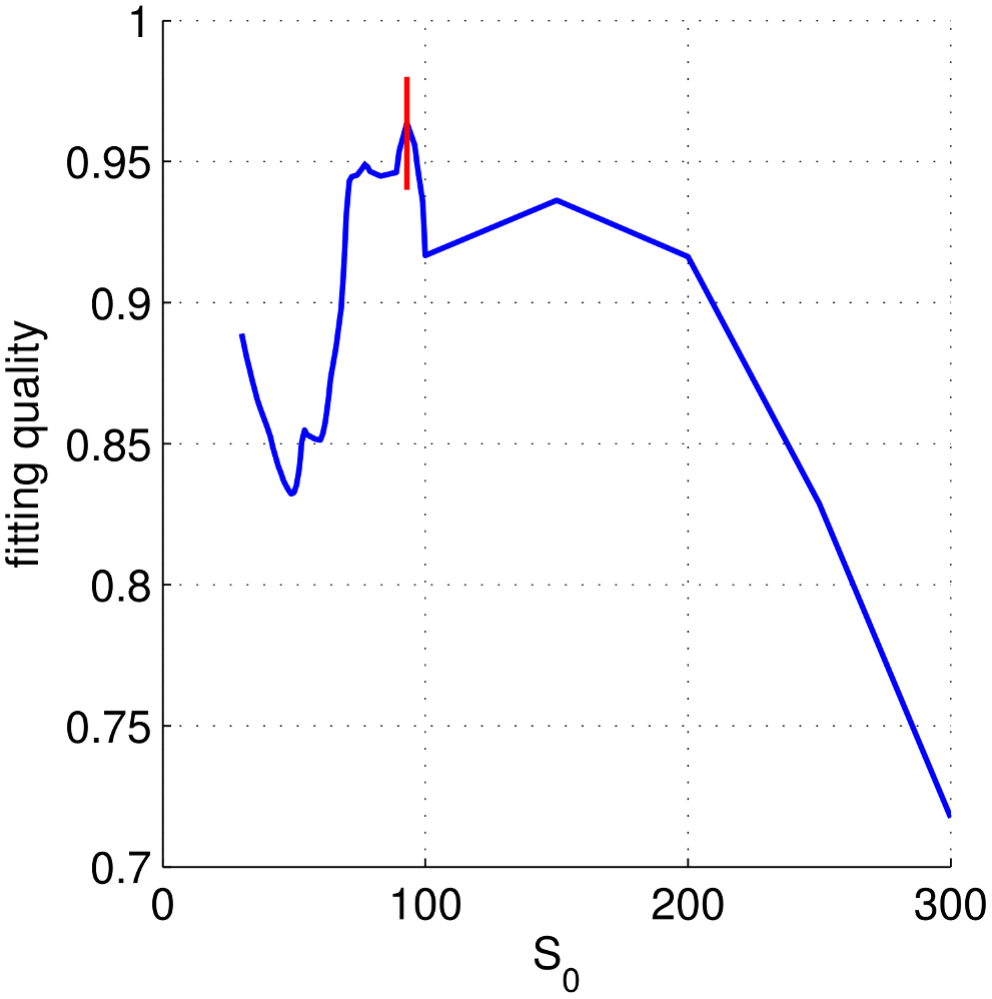
The selection of 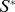 for Gowalla. The fitting quality as function of 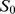. 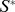 is defined as the position (argmax) of the maximum, which is 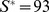 for Gowalla.

We choose 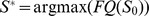 where 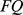 takes its maximum at the value of 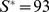, as shown in Figure 7.

### Spatially resolved monthly growth rates

For each location with integer ID 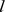, we extract a time series from 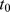 to 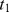 of the monthly population growth rate according to equation (1) as 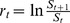, 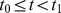 being the 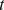
th month.

We calculate the autocorrelation function (ACF) from the time series 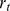 as
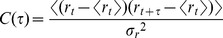
(9)where 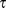 is the time lag and 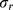 is the standard deviation of 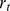.

### Superposition construction

To study superpositions we select all populations at locations with 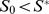. The randomized surrogate data set is created by shuffling these entries, and creating a time series from these shuffled entries as follows.

(1) From the set of populations with 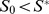 we select randomly a population and add up its initial population size 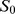, irrespective of its location. (2) We repeat (1) until the sum exceeds 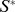. This results in a set of locations whose total initial population size equals or slightly exceeds 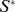. We call this set of locations one realization of a superposition. (3) For each realization we study the temporal development with respect to total populations size of the thereafter fixed selected locations. For each superposition we construct a time series, that is, the population growth rates in monthly resolution, from 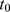 to 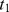. For this set of time series we obtain the ensemble averaged ACF.
